# Getting out of the box: the future of the UK donation after circulatory determination of death heart programme

**DOI:** 10.1016/j.eclinm.2023.102320

**Published:** 2023-11-19

**Authors:** John Onsy Louca, Alex Manara, Simon Messer, Marco Öchsner, David McGiffin, Isabel Austin, Eliza Bell, Savanna Leboff, Stephen Large

**Affiliations:** a University of Cambridge School of Clinical Medicine, Addenbrooke's Hospital, Hills Rd, Cambridge, CB2 0SP, UK; b The Intensive Care Unit, Southmead Hospital, North Bristol NHS Trust, Bristol, BS 10 5NB, UK; c Golden Jubilee Hospital, Agamermnon Street, Glasgow, G81 4DY, UK; d The Alfred and Monash University, Australia 55 Commercial Rd, Melbourne, VIC, 3004, Australia; e Royal Papworth Hospital Biomedical Campus, Cambridge, CB2 0AY, UK

**Keywords:** Heart transplantation, DCD, Donation after circulatory death, Donation after circulatory determination of death, Non-heart beating donation, taNRP, NRP, Thoraco-abdominal normothermic regional perfusion, In-situ perfusion, Ex-situ perfusion, Donor, Recipient

## Abstract

Heart failure imposes a significant burden on all health care systems and has a 5-year mortality of 50%. Heart transplantation and ventricular assist device (VAD) implantation are the definitive therapies for end stage heart disease, although transplantation appears to offer superior long-term survival and quality of life over VAD implantation. Transplantation is limited by a shortage in donor hearts, resulting in considerable waiting list mortality. Donation after circulatory determination of death (DCD) offers a significant uplift in the number of donors for heart transplantation. The outcomes both from the UK and internationally have been exciting, with outcomes at least as good as conventional donation after brain death (DBD) transplantation. Currently, DCD hearts are reperfused using ex-situ machine perfusion (ESMP). Whilst ESMP has enabled the development of DCD transplantation, it comes at significant cost, with the per run cost of approximately GBP £90,000. In-situ perfusion of the heart, otherwise known as thoraco-abdominal normothermic regional perfusion (taNRP) is cheaper, but there are ethical concerns regarding the potential to restore cerebral perfusion in the donor. We must determine whether there is any cerebral circulation during in-situ perfusion of the heart to ensure that it does not invalidate the diagnosis of death and potentially violate the dead donor rule. Besides this, there is a need for a randomised controlled trial to definitively determine whether taNRP offers any clinical advantages over ex-situ machine perfusion. This viewpoint article explores these issues in more detail.

**Key messages**

• Heart transplantation realises a significant improvement in quality and duration of life for those in end-stage, drug resistant and failing mechanical assisted devices heart failure. However, it is limited by the availability of donor hearts.
• Donation after circulatory determination of death (DCD) offers a possible 30% uplift in the number of heart transplants performed.
• In situ perfusion of the DCD heart, also known as thoraco-abdominal normothermic regional perfusion (ta-NRP) may have superior outcomes to ex-situ machine perfusion but there is a need for a randomised controlled trial in order to support or refute this statement.
• In situ perfusion raises other issues that need addressing before its more widespread implementation.

## The current state of heart transplantation

Heart transplantation is the definitive option for patients with end-stage heart failure, once medical therapy has been exhausted, and has been shown to significantly improve both quality and duration of life. Whilst ventricular assist devices (VADs) are an alternative option, their survival and quality of life (QoL) is not yet as good as transplantation and, importantly in the UK, they are only used as bridge to decision or bridge to transplantation.^1^^,^^2^ However, transplantation is limited by donor organ supply, resulting in nearly one third of waitlisted patients in the UK dying while awaiting heart transplantation.^3^ Moreover, improving life expectancy and mortality rates from severe heart disease (which was previously fatal) are increasing the number of potential heart recipients, adding further strain on the donor pool for heart transplantation. Indeed, the number of patients on the UK heart transplant waiting list has increased by over 50% in the last decade,^3^ with similar increases observed in the US and Europe.^4^^,^^5^ Recent strategies to bridge the supply-demand gap include using hearts from higher-risk donors (e.g. from hepatitis-positive organ donors^6^), implementing opt-out legislation, and using hearts recovered from donation after circulatory determination of death (DCD) donors. Although using hearts from hepatitis C-positive donors have shown encouraging results, it is unlikely to meaningfully increase transplant activity.^7^ Similarly, opt-out legislation has not yet shown evidence of consistent or meaningful increases in donation or transplantation rates.^8^ However, DCD has already significantly increased donor organ supply, with a possible 30% increase in the number of hearts transplanted (see Panel 1).^9^^,^^10^

*Panel 1* **Possible 30%.**

Several papers have cited the increase in transplantation activity due to DCD as 30%. This number is usually calculated as (number of DCD transplants/number of DBD transplants × 100). This calculation makes the reasonable assumption that DCD and DBD donors are distinct donor groups. However, currently in the UK, there is evidence that these groups overlap as a small number of eligible DBD donors may proceed via a DCD pathway to meet the wishes of the donor's family.

## Early challenges facing DCD transplantation

In the UK, all DCD donors are Maastricht category III donors i.e. those who die following a planned withdrawal of life-sustaining treatment. DCD heart transplantation was initially thought to be infeasible: the prolonged ischaemic times involved and the heart's intolerance to anoxia suggested that successful transplantation would be unlikely. DCD livers have a similar problem of poor ischaemic tolerance of the biliary tree, resulting in higher rates of ischaemic cholangiopathy.^11^^,^^12^

Therefore, unlike donation after brain death (DBD) donor hearts, DCD hearts require perfusion to ensure graft viability before transplantation.^13^ Reperfusion can either be performed inside the donor, otherwise known as thoraco-abdominal normothermic regional perfusion (taNRP), or outside the donor using ex-situ machine perfusion (ESMP). Both methods have been shown to be safe and effective.

## Ethical and other considerations in taNRP

A fundamental ethical principle in organ donation is respecting the dead donor rule^14^: the donor must be deceased before organ recovery commences, otherwise the organ recovery procedure would be the cause of death of the donor. The practice of taNRP raises both legal and ethical questions because it can challenge current definitions of death and the criteria to diagnose it. Some countries define death as the irreversible loss of circulatory function, meaning that re-establishing circulation using taNRP in the donor after the confirmation of death could be considered to invalidate the legal definition of death. In the UK, the definition of death has been based on the loss of brain functions (the permanent loss of capacity for consciousness, capacity to breathe and of brain stem functions) since the publication of the neurological criteria used to meet this definition in 1979.^15^ The Academy of Medical Royal Colleges’ 2008 code of practice for the diagnosis and confirmation of death published the circulatory criteria used to meet this same definition of death, effectively creating a unified definition of death that could be diagnosed using either circulatory or neurological criteria.^16^ It also made clear that after death was confirmed using circulatory criteria no interventions that could restore cerebral perfusion should be undertaken.

International consensus also favours the adoption a unified brain-based definition of death.^17^ This addresses the legal and ethical concerns raised by taNRP because the circulation that is relevant is the circulation to the brain: without circulation to the brain there can be no brain perfusion, and without brain perfusion there can be no brain function. If there is no restoration of brain circulation, the dead donor rule is respected.^18^ For taNRP it is therefore imperative to ensure that circulation to the brain is not restored by the procedure.^19^ Surgical techniques to prevent this have been described,^18^ and small studies in animals and case reports in humans suggest that these are effective.^19^^,^^20^ Recently, a prospective study with eight aNRP and two taNRP donors demonstrated no changes in pressure measured invasively within the circle of Willis. This is the first strong evidence that there is no cerebral blood flow in NRP.^21^ Larger scale human studies are however required for further reassurance before taNRP can be adopted as the technique of choice. Intraoperative studies using CT cerebral angiography during taNRP are currently planned in the UK and Spain. This reassurance. that there is no restoration of cerebral blood flow and that the dead donor rule is being respected, is essential before considering any potential benefits of taNRP.

taNRP is currently an unusual procedure and few retrieval surgeons have experience with it. As with any other medical intervention, adequate training is required before retrieval teams introduce this more complex form of DCD heart retrieval. Ultimately, all cardiothoracic retrieval teams need to ensure that cerebral circulation is excluded on every occasion, and that the warm ischaemic time to the heart is minimised.

There is also a need to understand how much information families would wish to receive about the technicalities of taNRP, and indeed of any other organ donation procedure. Little is known about the knowledge and attitudes of the public on this issue, particularly those registered on the organ donation register. Engagement of donor families and the public at large is required to ensure transparency during organ donation conversations and that consent to organ donation is truly informed. This will require qualitative research, possibly using structured interviews and/or questionnaires with thematic analysis of the responses.

## DCD heart transplantation is effective

DCD transplantation has demonstrated an overall survival similar to DBD transplantation (the current gold standard).^10^^,^^22^, ^23^, ^24^ The UK adopted a DCD heart programme in February 2015, shortly after the first adult DCD heart transplants in Australia.^25^ Many other countries have since initiated successful DCD programmes, including Austria, Belgium, the Netherlands, Spain, and the US.^10^^,^^23^ Both national and international observational studies report outcomes of DCD heart transplantation that are similar to DBD transplantation. Recently, our group collated the international experience of in situ (taNRP) perfusion of the DCD heart compared to contemporaneous DBD transplantation. In this study 136/157 of taNRP hearts used cold storage (CS). We demonstrated that survival was similar between the groups, as was the incidence of acute rejection. Interestingly, there was a lower rate of VAD usage in the group of recipients of in-situ perfused DCD hearts compared to the DBD group, suggesting a lower rate of primary graft dysfunction (PGD).^10^ Whilst encouraging, these figures are from an observational study and may be subject to bias due to the taNRP group having a younger donor age and greater proportion of male donors.

Results of DPP with ESMP have also been encouraging, with survival similar to DBD.^22^^,^^24^^,^^26^ However, DPP with ESMP is associated with higher use of temporary mechanical circulatory support and possibly higher incidence of PGD, although episodes of PGD seem to be milder and resolve quicker in the DPP group compared to the DBD group. We suggest that this difference is due to the different causes of graft dysfunction between the groups: in the DBD group, the mechanism may be either due to stress-induced takotsubo like cardiomyopathy caused by the Cushing response to neurological death or damage from prolonged cold ischaemia.^27^

However, in the DPP group, the mechanism is likely a result of warm ischaemia with subsequent cardiac stunning on reperfusion and distension of the right ventricle after withdrawal of life supporting treatment.^28^ We propose that the mechanisms of PGD in taNRP include this myocardial stunning seen as a result of warm ischaemia and possibly damage from prolonged warm ischaemia. It may be possible that this relatively short period of warm ischaemia followed by reperfusion in-situ acts as a form of preconditioning.

## Benefits of taNRP over DPP

Whilst outcomes for both taNRP and DPP with ESMP have been at least as good as DBD transplantation, there is a need to establish which method should be the mainstay of the national UK DCD programme. It has been suggested that hearts retrieved using taNRP have shown a markedly lower rate of acute rejection and a lower rate of PGD (Panel 2).^10^^,^^22^^,^^26^ However, these differences are likely to be complicated by bias, which may in part be due to the non-randomised small sample size in these studies. There is a need for an adequately powered randomised control trial (RCT) to confidently assess whether there are significant differences in outcomes for heart recipients with these methods.

*Panel 2* **ESMP vs taNRP–a false dichotomy?**

The DCD heart requires perfusion before transplantation. This can occur either ex-situ, in-situ or both. Therefore, taNRP and ESMP are not mutually exclusive and in our centre (Royal Papworth Hospital), most taNRP cases were preserved using ESMP. However, ESMP is associated with high costs and early data suggests that ESMP does not seem to offer any advantage over cold storage after taNRP. Indeed, most current practice with taNRP is with cold storage (CS). Therefore, we believe the choice comes down to whether it is best to perform direct procurement and preservation (DPP) with ESMP or taNRP with CS.

Whilst ESMP currently seems to offer little advantage to taNRP, future advancements in the field of ESMP such as prolonged perfusion times, better assessment of heart function and significantly cheaper costs may mean that taNRP and ESMP will be used in conjunction again.

Furthermore, a recent study has suggested taNRP could enable improved abdominal organ utilisation compared to direct procurement, albeit using a relatively small sample size.^29^ It is imperative that a RCT is conducted to better understand the differences between in-situ and ex-situ heart machine perfusion. Moreover, work from abdominal NRP has demonstrated superior outcomes and organ utilisation for both livers and kidneys compared to direct procurement.^30^^,^^31^^.^Therefore, taNRP may not only improve outcomes for the heart but also has the potential to improve abdominal organ outcomes and utilisation. taNRP may have unrealised potential to benefit both thoracic and abdominal transplant recipients and should be performed as the standard DCD organ recovery method. One risk is that complications (such as aortic dissection) can potentially compromise the use of all organs. However, this is uncommon, and we believe that the benefits outweigh the risks, with one reported recipient death caused by intraoperative complications out of 157 cases.^10^

taNRP appears to be more cost-effective than ESMP. Hearts recovered using taNRP can be preserved with (CS). Our previous study demonstrated that ESMP offered little advantage over conventional CS following taNRP.^10^ Similarly, in DBD transplantation with ex-situ preservation times less than 4 h and non-marginal hearts, it has been demonstrated that ESMP offers no advantage over hearts preserved with CS.^13^ Preservation with taNRP and CS costs less than £5,000, a fraction of the £350,000 of the TransMedics ESMP machine and per run cost of £90,000. This makes a DCD transplantation programme based on DPP less attractive, especially given the financial pressures on the NHS.

taNRP also enables better assessment of donor heart function in situ using transoesophageal echocardiography and pulmonary artery catheters.^32^ This is not possible when using ESMP where measurement of lactate is the only marker of heart function and suitability for transplantation. Our centre (Royal Papworth Hospital, Cambridge, UK) showed that arterial lactate levels and arteriovenous lactate differences do not correlate with the rate of PGD.^33^ Moreover, using lactate concentration as a biochemical marker for heart function was conducted with just 48 DBD donors, compromising its validity in DCD donation.^34^ This highlights the need for a reliable marker of organ viability. Also, taNRP enables assessment of heart function after the period of warm ischaemia and probable ventricular distension, allowing surgeons to assess and compare heart function before the withdrawal of life supporting treatment with that during NRP. This is not possible in DPP with ESMP, where surgeons can only use the pre-withdrawal echocardiograms to assess heart function. It is worth mentioning that there are tools that can better assess ex-situ hearts, such as pressure volume loop technology^32^ or analysis of the myocardial microcirculation.^35^ However, these tools are either not used widely clinically or are still only used experimentally.

Thus, taNRP offers many potential advantages over DPP. Therefore, the question remains as to how the UK should proceed with its DCD programme.

### Future directions: what next for the UK DCD programme?

The increasing impact of DCD heart transplantation in the UK is clear. In the year 2022, 215 heart transplants were performed, of which 55 were DCD, increasing activity by 34% when compared to DBD alone. We suggest that the following steps are required to inform a decision as to whether the UK should primarily adopt taNRP or DPP with ESMP.

1) Undertake further human studies to confirm the absence of brain circulation during taNRP.
2) Undertake a multicentre RCT to compare the cost-effectiveness and transplant outcomes of taNRP with those after ESMP. We suggest the inclusion of a DBD with CS cohort in the study to ensure that DCD transplant outcomes are non-inferior.
3) Conduct qualitative studies to establish how much detail donor families wish to receive regarding the technical operative procedures required for taNRP or indeed any other form of organ donation.
4) More rigorously analyse the impact of DCD heart transplant activity on waiting list mortality.

## Conclusions

DCD transplantation has resulted in a significant increase in heart transplantation across the UK. There is an urgent need to establish the most appropriate form of DCD heart retrieval to ensure that we are utilising donated organs appropriately to achieve best outcomes for recipients. If taNRP is to be recommended as the technique of choice, there is a need to for studies to demonstrate that taNRP respects the dead donor rule by not restoring brain blood flow or perfusion, and for a RCT to demonstrate that taNRP is indeed clinically superior to DPP.

## Contributors

John Onsy Louca—wrote the article. He is the guarantor on this article. He published the international experience of in-situ perfusion of the heart, published in eClinicalMedicine earlier this year.

Alex Manara—reviewed and critiqued the article. Alex Manara is an intensivist and prominent voice in the practice and ethics of organ donation both in the UK and abroad.

Simon Messer—reviewed the article. Consultant cardiac surgeon and responsible for pioneering the DCD heart transplantation programme.

Marco Ochsner—helped to write the article and provided critical comments on the final draft. He was co-author and performed the statistical analysis on the international experience of in-situ perfusion of the heart.

David McGiffin—reviewed and critiqued the article. Senior cardiothoracic surgeon, Alfred Health and Monash University.

Isobel Austin—performed the literature search and reviewed the article. A young doctor with an interest in transplantation.

Eliza Bell—patient involvement. A former heart transplant recipient with an interest in DCD transplantation. She helped with the initial conception for the paper and commented on the paper.

Savanna Leboff—reviewed and critiqued the article and has been a key member of our group for several months.

Stephen Large—reviewed the article. Consultant cardiac surgeon and responsible for pioneering the DCD heart transplantation programme.

## Declaration of interests

None of the authors have any conflicts of interest.
